# The role of backward cell migration in two-hit mutants’ production in the stem cell niche

**DOI:** 10.1371/journal.pone.0184651

**Published:** 2017-09-20

**Authors:** Audrey Bollas, Leili Shahriyari

**Affiliations:** 1 Department of Mathematics, The Ohio State University, Columbus, OH, United States of America; 2 Mathematical Biosciences Institute, The Ohio State University, Columbus, OH, United States of America; National Cancer Institute, UNITED STATES

## Abstract

It has been discovered that there are two stem cell groups in the intestinal crypts: central stem cells (CeSCs), which are at the very bottom of the crypt, and border stem cells (BSCs), which are located between CeSCs and transit amplifying cells (TAs). Moreover, backward cell migration from BSCs to CeSCs has been observed. Recently, a bi-compartmental stochastic model, which includes CeSCs and BSCs, has been developed to investigate the probability of two-hit mutant production in the stem cell niche. In this project, we improve this stochastic model by adding the probability of backward cell migration to the model. The model suggests that the probability of two-hit mutant production increases when the frequency of backward cell migration increases. Furthermore, a small non-zero probability of backward cell migration leads to the largest range of optimal values for the frequency of symmetric divisions and the portion of divisions at each stem cell compartment in terms of delaying 2-hit mutant production. Moreover, the probability of two-hit mutant production is more sensitive to the probability of symmetric divisions than to the rate of backward cell migrations. The highest probability of two-hit mutant production corresponds to the case when all stem cell’s divisions are asymmetric.

## Introduction

Studying stem cell dynamics is important for determining the origin of many diseases including cancer, and it may also suggest ways to obtain optimal treatments for these diseases. Stem cell therapy has been used for treating several diseases such as cancer [1]. Recently, scientists are trying to use umbilical cord stem cells (USCs), which are a source of mesenchymal stem cells (HUCMSCs) that promote tissue repair and modulate immune responses, to treat solid tumors. There is evidence that co-culture of rUSCs with Lewis lung carcinoma cells causes cancer cells to remain at the G0/G1 phase [2]. Saliently, in an in vivo study, the injection of rat umbilical cord SCs (rUSCs) could completely abolish rat mammary carcinomas [3].

Knowledge of stem cell division patterns such as their division and death rates, and the rate at which they divide symmetrically or asymmetrically can suggest ways to alter the stem cell niche in order to minimize the number of mutant cells in a tissue. Moran models, which assume a constant number of cells at each updating time step, are commonly used to study cell dynamics [4–9], because the number of cells in normal adult tissues stays approximately constant. For instance, it has been observed that the total number of cells in the normal intestinal and colon crypts stays approximately constant [10, 11], and because of the fairly simple structure of colon and intestinal crypts, many computational models have been developed to investigate cell dynamics in the crypts [12–19]. Additionally, several mathematical models have been designed to study the interplay between mutants and normal cells [20–29].

Tissue cells are categorized into two general groups, stem cells and non-stem cells. Stem cells are characterized by their ability to divide both symmetrically and asymmetrically. There are two types of stem cell symmetric divisions: proliferation (two newborn cells are SCs) and differentiation (two newborn cells are TAs). It has been suggested that stem cells in many tissues, including hair, blood, intestine, and brain [30], follow a bi-compartmental structure, which includes ‘border stem cells’ (BSCs) and ‘central stem cells’ (CeSCs). Lately, Ristma et al. [31] provided more details about how the two SC compartments, where each consists of approximately 7 SCs, work together to maintain a constant cell population in the mouse intestinal crypt. They observed that the BSCs, which are located between the transit amplifying cells (TAs) and the CeSCs, mostly differentiate in order to control the number of non-stem cells. Additionally, the CeSCs, which are located at the base of the crypt, mostly proliferate to control the total number of SCs. They also found that central stem cells can divide and migrate to the BSC compartment to replace cells in that region. Moreover, a small number of migrations of BSCs to CeCS was observed.

There are several mathematical models suggesting that stem cell symmetric division delays the production of two-hit mutants [6, 28, 32]. Two-hit mutant production is important because inactivation of tumor-suppressor genes resulting from double-hit mutations is one of the most common causes of carcinogenesis [33]. Recently, computational models have been designed to investigate the role of the bi-compartmental structure of the stem cell niche in the production and spread of mutants [7, 8]. We follow a model developed by Shahriyari and Komarova [7], which provides optimal division patterns in the SC niche in terms of minimizing the rate of double-hit mutations. This model does not consider the possibility of the migration of stem cells from the BSC compartment to the CeSC compartment. In the present work, we improve this model by incorporating this backward cell migration between the two stem cell compartments. We use both stochastic numerical simulations and analytical methods to investigate the role of backward cell migration in the probability of two-hit mutant production.

## Results

### Set-up

Recently, Shahriyari and Komarova [6] developed a bi-compartmental stochastic model, which includes two stem cell groups BSCs and CeSCs, for the stem cell niche, and they obtained the optimal parameters’ values, which delay two-hit mutant’s production. Here, we modify that model by adding the probability of backward cell migration from BSCs to CeSCs. In this Moran model, at each updating time step, two TA cells die and two stem cells divide to keep the total number of cells constant. We choose two cells in order to accommodate the symmetric division of stem cells. Note that two dead TAs could be replaced by divisions of TAs, however, here we only investigate cell dynamics in the stem cell niche. Therefore, we only focus on divisions in the SC niche. At each updating time step, with probability *σ*, two SCs will divide symmetrically (a differentiation is coupled with a proliferation), or with probability 1 − *σ* two SCs will divide asymmetrically. When SCs divide asymmetrically, one of newborn cells is a stem cell and the other one is a TA cell. In each division, we assume with a very small probability that a mutation occurs in one of the newborn children. In the asymmetric division, we assume with probability *ν* the mutation occurs in the stem daughter cell because of the immortal DNA strand hypothesis [34]. The hypothesis suggests that, upon asymmetric division, the DNA of a stem cell does not segregate randomly, but instead the daughter stem cell retains the parental strand. As a result, SCs pass mutations arising from errors in DNA replication on to their non-stem daughter cells. Although several groups support the immortal DNA strand hypothesis [35, 36], some scientists have a concern that there is no convincing experimental confirmation [37].

In this model, asymmetric divisions and differentiations can only happen in the BSC compartment because BSCs are close to the TA compartment. However, proliferations will happen in the CeSC compartment with probability *γ*, or in the BSC compartment with probability 1 − *γ*. When a proliferation occurs in the CeSC compartment, we assume a random cell migrates to the BSC compartment in order to keep the number of cells constant at each compartment. Since Ritsma et al. [31] observed backward cell migration from BSCs to CeSCs, we assume with probability *α* a random cell will migrate from BSCs to CeSCs when a BSC proliferates. When a random cell migrates from BSCs to CeSCs, then another migration happens from CeSCs to BSCs to keep the number of cells constant at each compartment.

**Fig 1:**
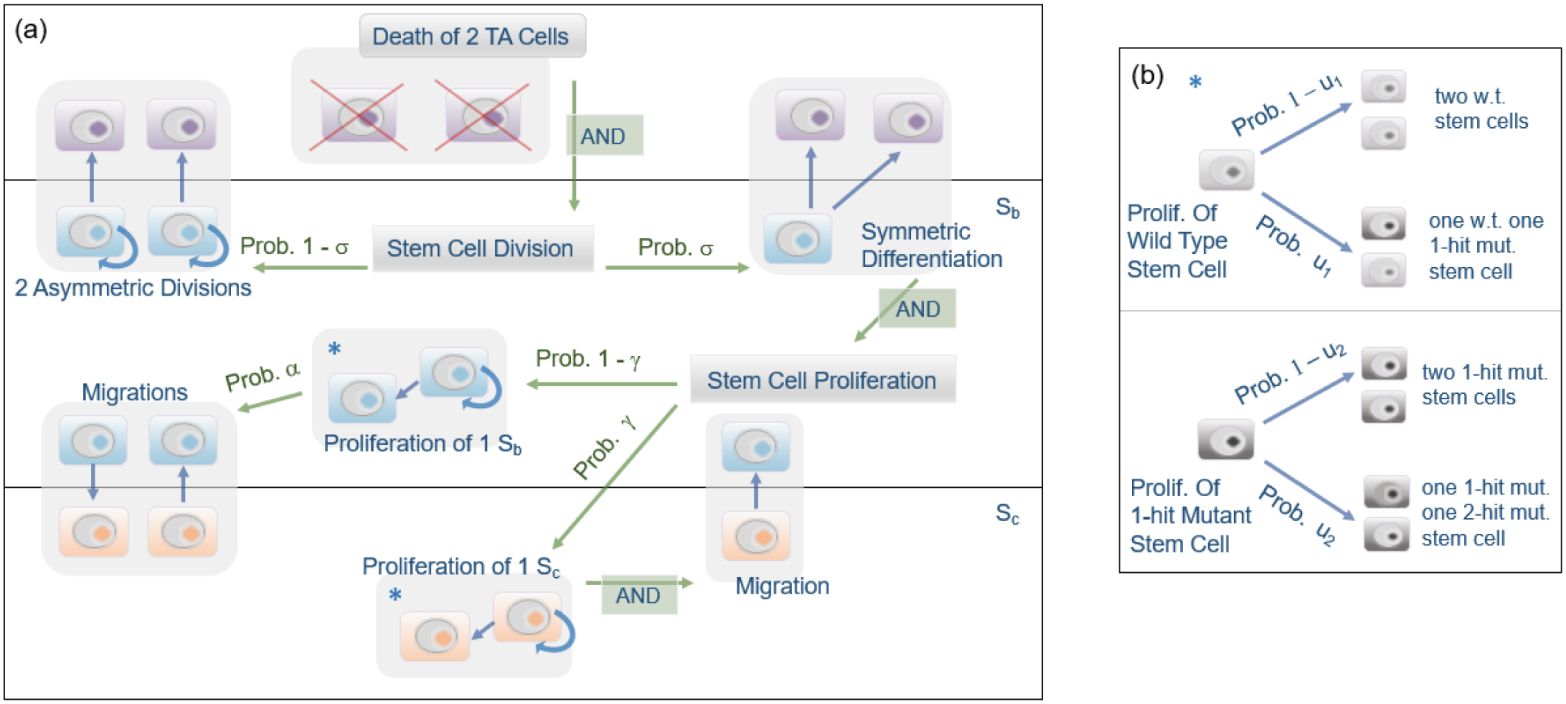
Schematic representation of the model. (a) The death of two TA cells initiates stem cell division in the *S*_*b*_ compartment to create two non-stem cells. Proliferation will occur in either of the two SC compartments to replace the differentiated cells. (b) Division tree for wild type and single-hit mutant stem cells.

We assume that the fitness of all wild-type stem cells is one, while the relative fitness of one-hit mutant cells is *r*. That means that when a division occurs in the CeSC (or BSC) compartment, with probability $\frac{re^{*}}{re^{*}+e}$ (or $\frac{rb^{*}}{rb^{*}+b}$) a one-hit mutant cell is chosen to divide, or with probability $\frac{e}{re^{*}+e}$ (or $\frac{b}{rb^{*}+b}$) a wild-type cell is chosen to divide. Symbols e* and *e* are respectively the number of one-hit CeSC mutants and wild-type CeSCs, and b* and *b* are respectively the number of one-hit BSC mutants and wild-type BSCs. We stop the algorithm as soon as the first two-hit mutant appears in the system. In summary, at each updating time step, we follow the below algorithm, which is presented in Fig 1, and the parameters of the model are given in Table 1.

**Table 1:** Model parameters.

Symbol	Description
*r*	fitness of 1-hit mutants
*σ*	Prob. of symmetric division
*γ*	Prob. of proliferation in CeSC
*α*	Prob. of migration from BSCs to CeSCs when a BSC proliferates
|*S*_*c*_|	Total number of CeSCs
|*S*_*b*_|	Total number of BSCs
*u*_1_	Prob. of a one-hit mutation in the division of a w.t. cell
*u*_2_	Prob. of a two-hit mutation in the division of a one-hit mutant
*ν*	Prob. of the SC daughter cell getting the mutation in an asymmetric division

#### The algorithm

Although we only keep track of divisions in the stem cell niche, the process begins with the death of two TA cells. These lost TA cells are replaced by either symmetric or asymmetric stem cell divisions in the BSC compartment; stem cells are randomly chosen to divide based on their fitness. With probability 1 − *σ* two asymmetric divisions will occur, and with probability *σ* a symmetric differentiation will occur. When there is a symmetric differentiation, further changes in the stem cell compartments must follow to maintain the constant cell population. With probability 1 − *γ* a second stem cell in the BSC compartment proliferates. And with probability *α* a migration between the two stem cell compartments will follow with one random cell from the BSC compartment migrating to the CeSC compartment, and one random CeSC migrating to the BSC compartment. The stem cell proliferation will occur in the CeSC compartment with probability *γ*, along with the migration of one CeSC to the BSC compartment.

Each time a wild type stem cell proliferates, there is a probability 1 − *u*_1_ that another wild type cell is created, and probability *u*_1_ that one of the daughter cells is a single-hit mutant. When a single-hit mutant cell proliferates, there is a probability 1 − *u*_2_ that another single-hit mutant is created and probability *u*_2_ that one of the daughter cells is a double-hit mutant. We chose similar values to those used in [38] for *u*_1_ and *u*_2_ to be able to compare the results. Note that there are some mathematical restrictions for these values in order to be in the tunneling regime, which aligns with reality. The assumption of the tunneling rate is that second-hit mutants are generated before one-hit mutants take over the entire tissue. In Moran models, in order to be in the tunneling regime the fixation rate must be smaller than the tunneling rate. For more details please see the Methods section.

If a mutation occurs when a stem cell divides asymmetrically, it is possible for the mutation to occur in the SC or in the differentiated daughter cell. We include the parameter *ν* to represent the probability of the mutation occurring in the stem cell.

### Tunneling rate

In order to calculate the probability of two-hit mutant production, we obtained tunneling rate—the rate at which the system of a given size produces two-hit mutants (assuming that one-hit mutants drift at relatively low levels). In other words, this is the rate at which the first double-hit mutant is produced before all cells become one-hit mutants. We found that the tunneling rate *R*_0 → 2_ satisfies the following formula; the detail of calculations is provided in the Methods section.

$$\begin{array}{ccc} R_{0\to 2} & = & -u_{1}\left(\sigma \gamma \left(1-y\right)+\left(\sigma \left(1-\gamma \right)+2\left(1-\sigma \right)\nu \right)\left(1-x\right)\right) \end{array}$$ (1)

Where *x* and *y* are the solutions of the following equations.

$$\begin{array}{ccc} 0 & = & \frac{\sigma \gamma }{S_{c}}\left(x+r\left(1-u_{2}\right)y^{2}-\left(r+1\right)y+\frac{1-\gamma }{\gamma }\alpha \left(x-y\right)\right), \end{array}$$ (2)

$$\begin{array}{ccc} 0 & = & \frac{r}{S_{b}}\left(\sigma \left[\left(1-\gamma \right)\left(1-u_{2}\right)x^{2}+\left(1-\gamma \right)\frac{\alpha }{r}\left(y-x\right)-\left(2-\gamma \right)x+1\right]-2\left(1-\sigma \right)u_{2}\nu x\right). \end{array}$$ (3)

We also ran stochastic simulations and compared the results of the simulations with the results of the formulas. In the numerical simulations, at each time step, two stem cells were chosen to divide following the above algorithm. We ran the algorithm until producing a two-hit mutant or reaching the maximum number of time-steps, *T*. Then, we checked if a two-hit mutant has been generated in *T* number of time steps. In order to obtain the probability of two-hit mutant production, we repeated this process for 100 times and obtained the ratio of the number of runs that lead to the production of a two-hit mutant to the total number of runs, which was 100. We repeated this procedure for 10 times to find the mean and standard deviation of the probabilities. In other words, we obtained the probability of two-hit mutant production for 10 times and then we calculated the mean and standard deviation of these 10 obtained probabilities. Note that we chose 100 and 10 because we get a reasonable standard deviation in a fairly fast computing time period using our limited computational power.

### Backward cell migration increases the probability of 2-hit mutant production

**Fig 2:**
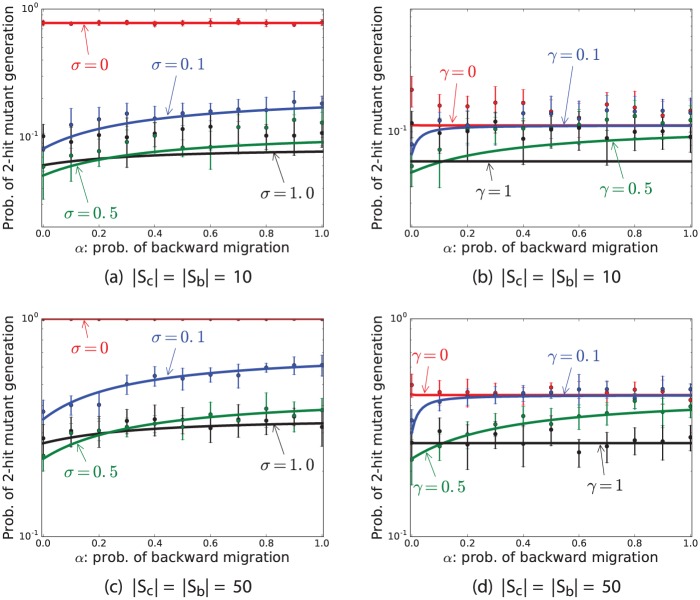
Probability of 2-hit mutant production as a function of *α*. This figure represent the mean and standard deviations of results of simulations (points and bars) and results of formula (solid lines) for the probability of 2-hit mutant production as a function of *α*, the probability of backward cell migration. The parameters are *ν* = 0.5, *u*_1_ = 10^−4^, *u*_2_ = 0.002, *r* = 1, *t* = 1000, and (a) *γ* = 0.5 and (b) *σ* = 0.5.

Although the probability of 2-hit mutant production is an increasing function of *α* when *γ* > 0, the probability of double-hit mutant production is not very sensitive to *α*, when *α* > 0 (Fig 2). When *γ* = 1, no cell divides symmetrically in the BSC compartment. Therefore, when *γ* = 1, the probability of 2-hit mutant production is independent of *α*, because backward migration occurs when border SCs divide symmetrically. When *γ* = 0, all stem cell divisions occur in the BSC compartment, and no division occurs in the CeSC compartment. In this case the probability of 2-hit mutant production is a decreasing function of *α*, because when a mutant migrates to the CeSC it will never have a chance to divide. In other words, when *γ* = 0 and *α* is a high number, there is a high probability that one-hit BSC mutants migrate to the CeSC compartment and never divide to generate a double-hit mutant. However, when 0 < *γ* < 1, the probability of two-hit mutant production is an increasing function of *α*. This means when stem cells are able to divide symmetrically in both SC compartments, the optimal value for *α* is zero in terms of delaying 2-hit mutant production. Note, in general the probability of two-hit mutant production is not very sensitive to *α* as long as *α* is not zero (Fig 2).

### The variation of *α* changes the optimal values for *σ* and *γ*

**Fig 3:**
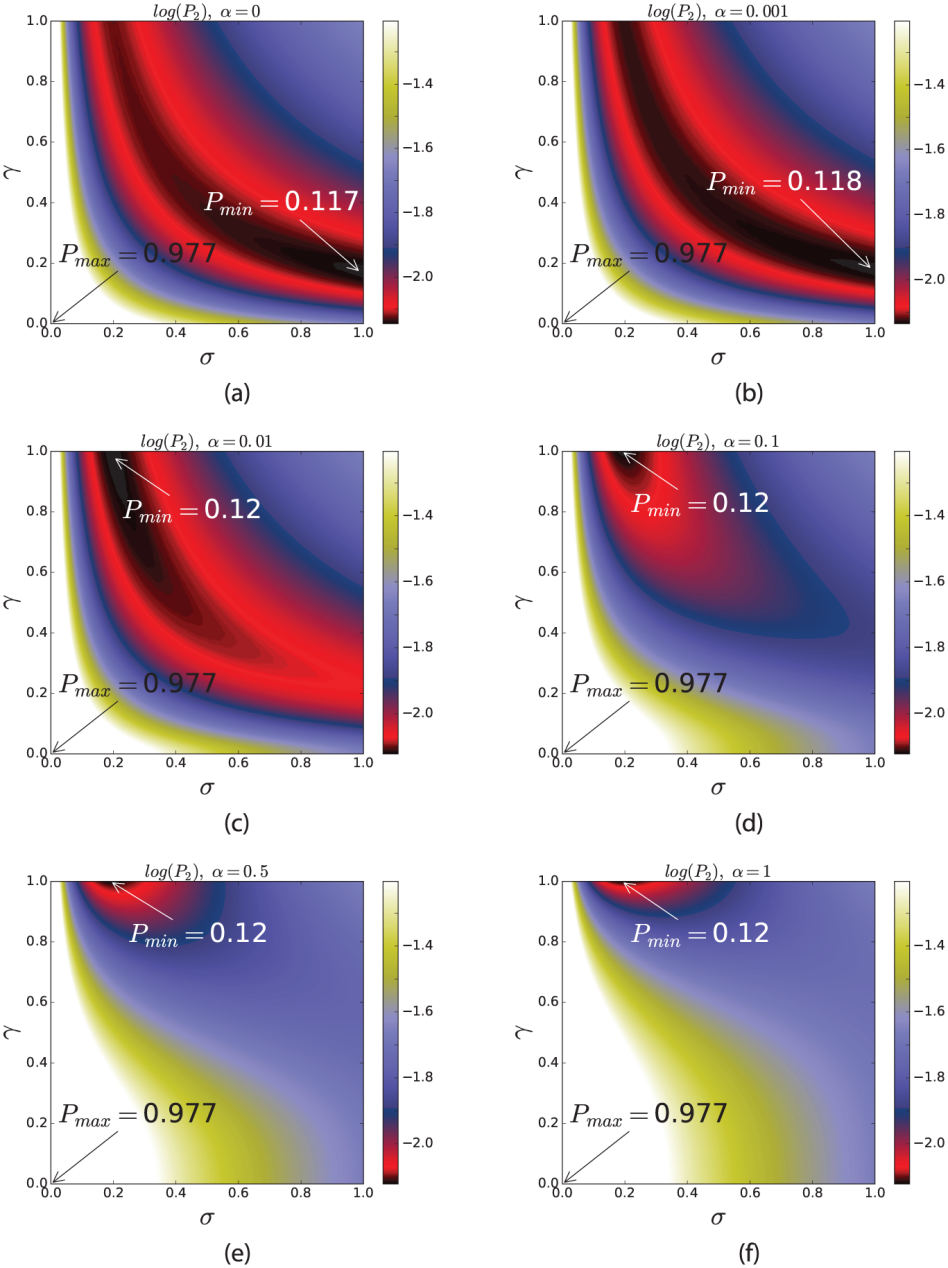
Probability of 2-hit mutant production as a function of *σ* and *γ*. This figure the results of formula for the probability of 2-hit mutant production (*P*_2_) as a function of *σ* and *γ*. The parameters are |*S*_1_| = |*S*_2_| = 25, *ν* = 0.5, *u*_1_ = 10^−4^, *u*_2_ = 0.002, *r* = 1, *t* = 1000, and (a) *α* = 0, (b) *α* = 0.001, (c) *α* = 0.01, (d) *α* = 0.1, (e) *α* = 0.5, and (f) *α* = 1.

Fig 3 shows a trend of changes in the optimal values for *γ* and *σ* when *α* varies. This figure reveals that a small non-zero percentage of backward cell migration leads to a higher range (or a higher interval) for the optimal values of *σ* and *γ* in terms of delaying two-hit mutant production. In other words, the regimes in which probability of two-hit mutant production is small, i.e. the dark areas, are much larger for *α* = 0.001 than for any other values of *α* in Fig 3. However, *α* = 1 corresponds to the worst case, which gives the largest intervals for the values of *σ* and *γ* causing a high probability of double-hit mutant production.

Fig 3 also shows that the optimal case corresponds to the parameter values, *γ* ≈ 0.3, *σ* = 1, and *α* = 0. In other words, the probability of two-hit mutant production is minimized when SCs only divide symmetrically while around 70% of SC proliferations happen in the BSC compartment, and no migration occurs from BSCs to CeSCs.

### Symmetric division delays two-hit mutant production when proliferations mostly occur in the BSC compartment and *α* > 0.2

**Fig 4:**
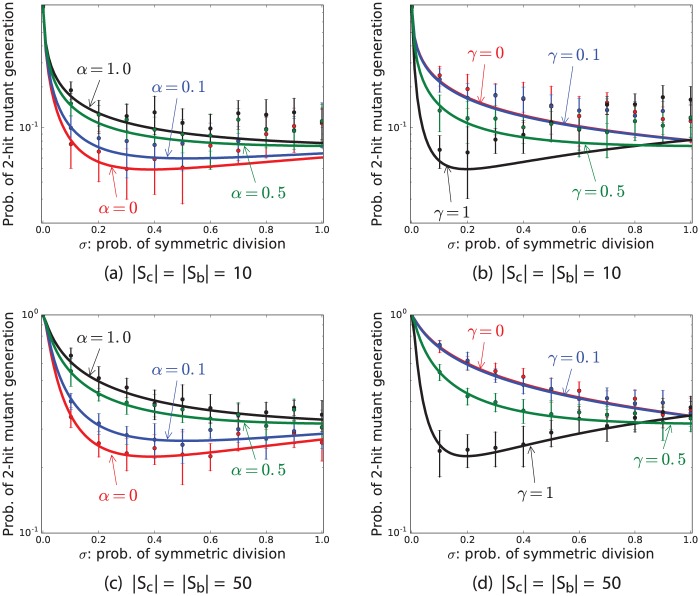
Probability of 2-hit mutant production as a function of *σ*. This figure represent the mean and standard deviations of results of simulations (points and bars) and results of formula (solid lines) for the probability of 2-hit mutant production as a function of *σ*, the probability of symmetric stem cell divisions. The parameters are *ν* = 0.5, *u*_1_ = 10^−4^, *u*_2_ = 0.002, *r* = 1, *t* = 1000, and (a,c) *γ* = 0.5 and (b,d) *α* = 0.5.

Stochastic simulations and analytic calculations show that the probability of two-hit mutant production is crucially affected by the frequency of symmetric divisions (parameter *σ*). Fig 2(a) and 2(c) show that when the probability of backward cell migration, *α*, is more than 0.2 and *γ* = 0.5, then the minimum of the probability of 2-hit mutant production corresponds to *σ* = 1. Moreover, when *α* > 0.2, the probability of two-hit mutant production decreases when *σ* increases (Fig 4). However, when *γ* = 0.5 and there are no backward cell migration in the niche (*α* = 0), then the optimal value for *σ* is around 0.3 in terms of delaying two-hit mutant production (Fig 4). Importantly, the probability of two-hit mutant production maximized when stem cells divide only asymmetrically (*σ* = 0) for all values of *α* (Fig 4). More precisely, if at least 50% of times mutations occur in the stem cell offspring in the asymmetric divisions (*ν* ≥ 0.5) and stem cells only divide asymmetrically, then the probability of two-hit mutant production is very high (Fig 4).

### When all SC proliferations occur in the CeSC compartment, i.e. *γ* = 1, then SCs should divide both symmetrically and asymmetrically, but mostly asymmetrically to delay two-hit mutant production

Fig 4(b) and 4(d) show that the minimum value of the probability of double-hit mutant production corresponds to *σ* ≈ 0.2, when *α* = 0.5 and *γ* = 1. When *γ* = 1, then no stem cell proliferation occurs in the BSC compartment, and all stem cell proliferations happen in the CeSC compartment. Since mutants in the CeSC compartment have a higher chance of survivability, the probability of production of two-hit mutants minimizes when the rate of proliferation in the CeSC compartment decreases. Thus, when *γ* = 1, in order to minimize the number of divisions in the CeSC compartment, *σ*, probability of symmetric division, should be small. Note that the maximum of the probability of two-hit mutant production always corresponds to *σ* = 0, because when stem cells divide purely asymmetrically, mutant cells will never have a chance to differentiate and be removed from the stem cell niche. However, when stem cells divide symmetrically, there is always a chance that mutant cells differentiate into two mutant non-stem cells and become removed from the stem cell niche.

### Probability of two-hit mutant production is not sensitive to the percentage of cells at each compartment

**Fig 5:**
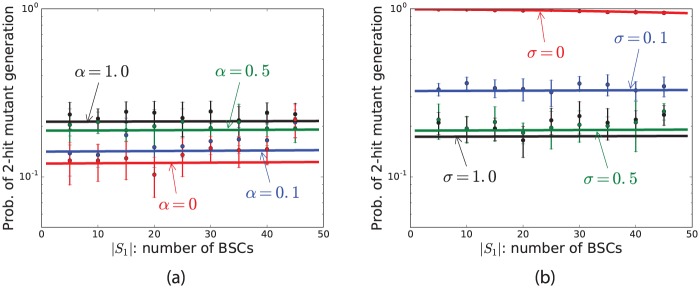
Probability of 2-hit mutant production varying |*S*_*b*_|. This figure represent the mean and standard deviations of results of simulations (points and bars) and results of formula (solid lines) for the probability of 2-hit mutant production as a function of |*S*_*b*_|, the number of stem cells in the BSC compartment. The parameters are *N* = 50, *ν* = 0.5, *u*_1_ = 10^−4^, *u*_2_ = 0.002, *r* = 1, *t* = 1000, *γ* = 0.5 and (a) *σ* = 0.5 and (b) *α* = 0.5.

Fig 5 represents the results for varying the number of cells in each compartment while the total number of cells is fixed (*N* = 50). For example, in this figure, when |*S*_*b*_| = 5, |*S*_*c*_| = 45. This figure indicates that the probability of two-hit mutant production is not very sensitive to the percentage of cells at each compartment. Additionally, like other figures, it clearly shows that when *σ* = *γ* = 0.5, the probability of two-hit mutant production is an increasing function of *α*. Moreover, when *α* = *γ* = 0.5, the probability of two-hit mutant production is a decreasing function of *σ*. In general, Fig 5 indicates that the probability of two-hit mutant production is not very sensitive to the percentage of cells at each compartment, and it is more sensitive to *σ* than to *α*.

Note that in the analytical calculations, we assume the number of cells is large in order to be able to derive the formulas. More precisely, we simplify the transition probabilities by assuming the number of mutants is much less than the total number of cells. This simplification leads to a noticeable difference between the result of the formulas and numerical simulations when the number of cells is small, particularly when the number of CeSCs is small (See Fig 5). However, the trend stays the same, and therefore the results do not change.

### The immortal DNA strand hypothesis

It has been hypothesized that, in asymmetric divisions, the DNA of a SC does not segregate randomly, but instead the stem daughter cell retains a distinct template set of DNA strands (called the parental strand). Therefore, SCs pass mutations arising from errors in DNA replication onto their TA daughters, which soon terminally differentiate to minimize the probability of mutants’ production in the stem cells.

**Fig 6:**
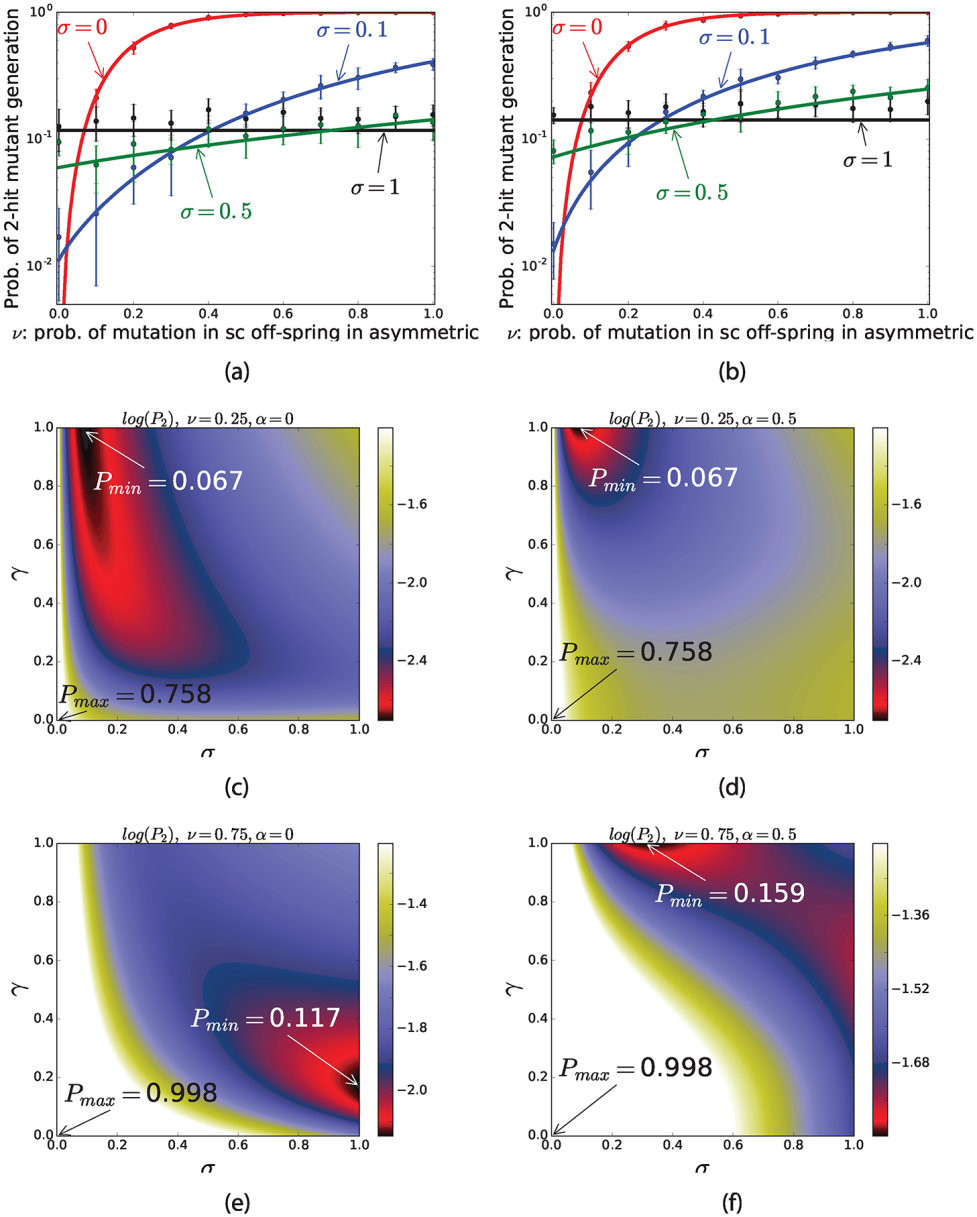
The effect of immortal DNA strand hypothesis on probability of 2-hit mutant production. The sub-figure (a) shows the probability of 2-hit mutant production as a function of *ν*, points and bars are results of simulations and solid lines represents the results of formula. The sub-figure(b-d) indicate the results of formula for the probability of 2-hit mutant production as a function of *σ* and *γ*. The parameters are *u*_1_ = 10^−4^, *u*_2_ = 0.002, *r* = 1, *t* = 1000, (a-b) |*S*_1_| = |*S*_2_| = 20, (a) *α* = 0, (b) *α* = 0.5, (c-f) |*S*_1_| = |*S*_2_| = 20, (c-d) *ν* = 0.25 (c) *α* = 0 (d) *α* = 0.5, (e-f) *ν* = 0.75, (e) *α* = 0, (f) *α* = 0.5.

We incorporate this mechanism into our model by introducing a parameter *ν*, which quantifies the probability of a mutation happing in the stem daughter cell of an asymmetrically dividing stem cell rather than in its TA offspring. Figs 2–5 show the results for *ν* = 0.5, which corresponds to a complete symmetry between stem and TA offspring (i.e. relaxing the assumption of immortal DNA strand hypothesis). Fig 6 presents the probability of double-hit mutant generation as a function of *ν*. In the case of *ν* = 0, the asymmetric division is optimal, because no mutations can occur in SC daughter cells when SCs divide asymmetrically, while CSs have a chance to acquire mutations in symmetric divisions. On the other hand, if *ν* > 0.5, then symmetric division is optimal. However, if 0 < *ν* < 0.5, then a mixture of symmetric and asymmetric divisions comprises the optimal strategy. The counter plots in Fig 6 show how the optimal regimes move toward *σ* = 1 when *ν* is increasing.

## Discussion

One of the most common patterns of carcinogenesis is the inactivation of tumor suppressor genes. Knudson suggested that two hits (mutation in both alleles) are required to inactivate a tumor suppressor gene [33, 39]. Many cell dynamics models have been developed to investigate the process of two-hit mutants’ production to answer some of the following questions. What is the optimal division pattern that minimizes the probability of two-hit mutant productions? Are healthy tissues following the optimal patterns? Are the optimal patterns violated in abnormal tissues like tumors? Is there anyway that we can alter the tissue’s structure to minimize the size of tumors? Lately, mathematical models show that stem cell symmetric divisions delay 2-hit mutant productions [6, 28, 32], and importantly it has been observed that adult stem cells in normal tissues divide mostly symmetrically [40]. Here, the model indicates that a small number of backward SC migrations, which have been experimentally observed, enlarge the interval of optimal values for other parameters. More precisely, the probability of two-hit mutant production is not very sensitive to a small perturbation in the optimal values of other parameters like the rate of symmetric divisions or CeSC’s proliferation rates when there are a small number of backward migrations in the SC niche.

The model also predicts that if *γ* < 1 and *α* > 0, then symmetric division delays the production of two-hit mutants. In other words, if the probability of CeSCs’s proliferation is less than one, and there is a migration from CeSCs to BSCs, then SCs should divide mostly symmetrically to delay two-hit mutant production. Ritsma et al. [31] observed backward cell migration from BSCs to CeSCs, i.e. *α* > 0. They also estimated that the probability of proliferation of CeSCs to be 0.3, i.e. 0 < *γ* < 1. Importantly, it has been observed that the intestinal stem cells divide mostly symmetrically, and asymmetric stem cell divisions rarely occur [40].

The model suggests that backward cell migration very slightly increases the chance of two-hit mutant productions when other parameter values are fixed. Importantly, a small non-zero value for *α*, the probability of backward cell migration, leads to the largest interval of optimal values for *σ*, the frequency of symmetric divisions, and *γ*, the probability of CeSC’s proliferations, in terms of delaying 2-hit mutant production. Although a small non-zero probability of backward cell migration expands the range of optimal values for *σ* and *γ*, a high probability of backward cell migration enlarge the intervals for values of *σ* and *γ* that lead to a high probability of two-hit mutant production. Furthermore, the frequency of SC symmetric and asymmetric divisions has a much higher effect on the probability of two-hit mutant production than the probability of backward cell migration. Moreover, the rate of double-hit mutant production is maximized when stem cells divide only asymmetrically.

## Materials and methods

### Transition probabilities

In order to calculate the probability of a two-hit mutant production, we found all other non-zero transition probabilities, which are the probabilities of moving from e* mutants in the CeSC compartment and b* mutants in the BSC compartment to another state in the model. We define *S*_*b*_ as the total number of stem cells in the BSC compartment and *S*_*c*_ as the total number of stem cells in the CeSC compartment. We denote the number of wild-type CeSCs by *e* = *S*_*c*_ − e* and the number of wild-type BSCs by *b* = *S*_*b*_ − b*. The transition probabilities of the model are as follows.

The probability that the number of mutant *S*_*b*_ cells increases by one while the number of mutant *S*_*c*_ cells does not change:

$$\begin{array}{ccc} P_{\left(e^{*},b^{*}\right)\to \left(e^{*},b^{*}+1\right)} & = & \underset{\text{Diff.}\;\text{of}\;\text{a}\;\text{w.t.}\;S_{b}}{\underset{︸}{\sigma \frac{b}{rb^{*}+b}}}[\left(1-\gamma \right)(\underset{\begin{array}{c} \text{Prolif.}\;\text{of}\;\text{w.t.}\;S_{b}\;\text{into}\\ \;\text{one}\;\text{w.t.}\;\text{and}\;\text{one}\;\text{mut.} \end{array}}{\underset{︸}{\frac{b-1}{rb^{*}+\left(b-1\right)}u_{1}}}(\overset{\text{No}\;\text{migration}}{\overset{︷}{\left(1-\alpha \right)}}\\ & & +\overset{\text{Migrations:}\;\text{a}\;\text{w.t./mut.}\;S_{b}\;\text{to}\;S_{c}\;\text{and}\;\text{a}\;\text{w.t./mut.}\;S_{c}\;\text{to}\;S_{b}}{\overset{︷}{\alpha \frac{\left(b^{*}+1\right)e^{*}+\left(b-1\right)e}{S_{b}S_{c}}}})+\underset{\text{Prolif.}\;\text{of}\;\text{mut.}\;S_{b}\;\text{into}\;\text{two}\;\text{mut.}}{\underset{︸}{\frac{rb^{*}}{rb^{*}+\left(b-1\right)}\left(1-u_{2}\right)}}\\ & & \times (\overset{\text{No}\;\text{migration}}{\overset{︷}{\left(1-\alpha \right)}}+\overset{\text{Migrations:}\;\text{w.t./mut.}\;S_{b}\;\text{to}\;S_{c}\;\text{and}\;\text{a}\;\text{w.t./mut.}\;S_{c}\;\text{to}\;S_{b}}{\overset{︷}{\alpha \frac{\left(b^{*}+1\right)e^{*}+\left(b-1\right)e}{S_{b}S_{c}}}}))\\ & & +\gamma \left(\underset{\begin{array}{c} \text{Prolif.}\;\text{of}\;\text{a}\;\text{w.t.}\;S_{c}\;\text{into}\;\text{one}\;\text{w.t.}\\ \;\text{and}\;\text{one}\;\text{mut.}\;\text{and}\;\text{mig.}\;\text{of}\;\text{a}\;S_{c}\;\text{mut.}\;\text{to}\;S_{b} \end{array}}{\underset{︸}{\frac{e}{re^{*}+e}u_{1}\frac{e^{*}}{S_{c}}}}+\overset{\text{Prolif.}\;\text{of}\;\text{mut.}\;S_{c}\;\text{into}\;\text{two}\;\text{mut.}\;\text{and}\;\text{mig.}\;\text{of}\;S_{c}\;\text{mut.}\;\text{to}\;S_{b}}{\overset{︷}{\frac{re^{*}}{re^{*}+e}\left(1-u_{2}\right)\frac{e^{*}-1}{S_{c}}}}\right)]+\underset{\begin{array}{c} \text{two}\;\text{asym.}\;\text{w.t.}\;S_{b}\;\text{divisions}\\ \;\text{with}\;\text{mut.}\;\text{in}\;S_{b} \end{array}}{\underset{︸}{2\left(1-\sigma \right)\frac{b}{rb^{*}+b}u_{1}\nu }}\\ & & \approx \sigma \frac{1-\gamma }{S_{b}}\left(rb^{*}\left(1-u_{2}\right)+S_{b}u_{1}\right)+\sigma \gamma u_{1}\frac{e^{*}}{S_{c}}+2\left(1-\sigma \right)u_{1}\nu \end{array}$$ (4)

The probability that the number of mutant *S*_*b*_ cells decreases by one while the number of mutant *S*_*c*_ cells stays the same:

$$\begin{array}{ccc} p_{\left(e^{*},b^{*}\right)\to \left(e^{*},b^{*}-1\right)} & = & \underset{\text{Diff.}\;\text{of}\;\text{w.t.}\;S_{b}}{\underset{︸}{\sigma \frac{rb^{*}}{rb^{*}+b}}}[\left(1-\gamma \right)\underset{\text{Prolif.}\;\text{of}\;\text{a}\;\text{w.t.}\;S_{b}\;\text{into}\;\text{two}\;\text{w.t.}}{\underset{︸}{\frac{b}{r(b^{*}-1)+b}\left(1-u_{1}\right)}}(\overset{\text{No}\;\text{migration}}{\overset{︷}{\left(1-\alpha \right)}}\\ & & +\overset{\text{Migrations:}\;\text{a}\;\text{w.t./mut.}\;S_{b}\;\text{to}\;S_{c}\;\text{and}\;\text{a}\;\text{w.t./mut.}\;S_{c}\;\text{to}\;S_{b}}{\overset{︷}{\alpha \frac{\left(b^{*}-1\right)e^{*}+\left(b+1\right)e}{S_{b}S_{c}}}})+\\ & & \underset{\text{Prolif.}\;\text{of}\;\text{a}\;\text{w.t.}\;S_{c}\;\text{into}\;\text{two}\;\text{w.t.}\;\text{and}\;\text{mig.}\;\text{of}\;\text{w.t.}\;S_{c}\;\text{to}\;S_{b}}{\underset{︸}{\gamma \frac{e}{re^{*}+e}\left(1-u_{1}\right)\frac{e}{S_{c}}}}]\approx \sigma \frac{rb^{*}}{S_{b}}\left(1-u_{1}\right) \end{array}$$ (5)

The probability that the number of mutant *S*_*b*_ cells decreases by one while the number of mutant *S*_*c*_ cells increases by one:

$$\begin{array}{ccc} p_{\left(e^{*},b^{*}\right)\to \left(e^{*}+1,b^{*}-1\right)} & = & \underset{\text{Diff.}\;\text{of}\;\text{a}\;\text{mut.}\;S_{b}}{\underset{︸}{\sigma \frac{rb^{*}}{rb^{*}+b}}}[\left(1-\gamma \right)(\underset{\text{Prolif.}\;\text{of}\;\text{a}\;\text{w.t.}\;S_{b}\;\text{to}\;\text{one}\;\text{w.t.}\;\text{and}\;\text{one}\;\text{mut.}}{\underset{︸}{\frac{b}{r(b^{*}-1)+b}u_{1}}}\overset{\begin{array}{c} \text{Migrations:}\;\text{a}\;\text{mut.}\;S_{b}\;\text{to}\;S_{c}\\ \;\text{and}\;\text{a}\;\text{w.t.}\;S_{c}\;\text{to}\;S_{b} \end{array}}{\overset{︷}{\alpha \frac{b^{*}}{S_{b}}\frac{e}{S_{c}}}}\\ & & +\underset{\text{Prolif.}\;\text{of}\;\text{a}\;\text{mut.}\;S_{b}\;\text{into}\;\text{two}\;\text{mut.}}{\underset{︸}{\frac{r(b^{*}-1)}{r(b^{*}-1)+b}\left(1-u_{2}\right)}}\overset{\text{Migrations:}\;\text{a}\;\text{mut.}\;S_{b}\;\text{to}\;S_{c}\;\text{and}\;\text{a}\;\text{w.t.}\;S_{c}\;\text{to}\;S_{b}}{\overset{︷}{\alpha \frac{b^{*}}{S_{b}}\frac{e}{S_{c}}}})\\ & & +\gamma (\overset{\text{Prolif.}\;\text{of}\;\text{a}\;\text{mut.}\;S_{c}\;\text{into}\;\text{two}\;\text{mut.}\;\text{and}\;\text{mig.}\;\text{of}\;\text{w.t.}\;S_{c}\;\text{to}\;S_{b}}{\overset{︷}{\frac{re^{*}}{re^{*}+e}\left(1-u_{2}\right)\frac{e}{S_{c}}}}+\underset{\text{Prolif.}\;\text{of}\;\text{a}\;\text{w.t.}\;S_{c}\;\text{into}\;\text{one}\;\text{w.t.}\;\text{and}\;\text{one}\;\text{mut.}\;\text{and}\;\text{mig.}\;\text{of}\;\text{a}\;\text{w.t.}\;S_{c}\;\text{to}\;S_{b}}{\underset{︸}{\frac{e}{re^{*}+e}u_{1}\frac{e}{S_{c}}}})]\\ & & +\underset{\text{Diff.}\;\text{of}\;\text{a}\;\text{w.t.}\;S_{b}}{\underset{︸}{\sigma \frac{b}{rb^{*}+b}}}\left(1-\gamma \right)\overset{\text{Prolif.}\;\text{of}\;\text{a}\;\text{w.t.}\;S_{b}\;\text{into}\;\text{two}\;\text{w.t.}}{\overset{︷}{\frac{b-1}{rb^{*}+\left(b-1\right)}\left(1-u_{1}\right)}}\underset{\begin{array}{c} \text{Migrations:}\;\text{a}\;\text{mut.}\;S_{b}\;\text{to}\;S_{c}\\ \;\text{and}\;\text{a}\;\text{w.t.}\;S_{c}\;\text{to}\;S_{b} \end{array}}{\underset{︸}{\alpha \frac{b^{*}}{S_{b}}\frac{e}{S_{c}}}}\\ & & \approx \sigma \left(1-\gamma \right)\left(1-u_{1}\right)\alpha \frac{b^{*}}{S_{b}} \end{array}$$ (6)

The probability that the number of mutant *S*_*b*_ cells stays the same while the number of mutant *S*_*c*_ cells decreases by one:

$$\begin{array}{ccc} p_{\left(e^{*},b^{*}\right)\to \left(e^{*}-1,b^{*}\right)} & = & \underset{\text{Diff.}\;\text{of}\;\text{a}\;\text{mut.}\;S_{b}}{\underset{︸}{\sigma \frac{rb^{*}}{rb^{*}+b}}}[\left(1-\gamma \right)\underset{\text{Prolif.}\;\text{of}\;\text{a}\;\text{w.t.}\;S_{b}\;\text{into}\;\text{two}\;\text{w.t.}}{\underset{︸}{\frac{b}{r(b^{*}-1)+b}\left(1-u_{1}\right)}}\overset{\begin{array}{c} \text{Migrations:}\;\text{a}\;\text{w.t.}\;S_{b}\;\text{to}\;S_{c}\\ \;\text{and}\;\text{a}\;\text{mut.}\;S_{c}\;\text{to}\;S_{b} \end{array}}{\overset{︷}{\alpha \frac{b+1}{S_{b}}\frac{e^{*}}{Sc}}}\\ & & +\gamma \underset{\text{Prolif.}\;\text{of}\;\text{a}\;\text{w.t.}\;S_{c}\;\text{into}\;\text{two}\;\text{w.t.}\;\text{and}\;\text{mig.}\;\text{of}\;\text{a}\;\text{mut.}\;S_{c}\;\text{to}\;S_{b}}{\underset{︸}{\frac{e}{re^{*}+e}\left(1-u_{1}\right)\frac{e^{*}}{S_{c}}}}]\approx 0 \end{array}$$ (7)

The probability that the number of mutant *S*_*b*_ cells increases by one while the number of mutant *S*_*c*_ cells decreases by one:

$$\begin{array}{ccc} p_{\left(e^{*},b^{*}\right)\to \left(e^{*}-1,b^{*}+1\right)} & = & \underset{\text{Diff.}\;\text{of}\;\text{a}\;\text{mut.}\;S_{b}}{\underset{︸}{\sigma \frac{rb^{*}}{rb^{*}+b}}}\left(1-\gamma \right)(\underset{\begin{array}{c} \text{Prolif.}\;\text{of}\;\text{a}\;\text{w.t.}\;S_{b}\;\text{into}\\ \;\text{one}\;\text{w.t.}\;\text{and}\;\text{one}\;\text{mut.} \end{array}}{\underset{︸}{\frac{b}{r(b^{*}-1)+b}u_{1}}}\overset{\begin{array}{c} \text{Migrations:}\;\text{a}\;\text{w.t.}\;S_{b}\;\text{to}\;S_{c}\\ \text{and}\;\text{a}\;\text{mut.}\;S_{c}\;\text{to}\;S_{b} \end{array}}{\overset{︷}{\alpha \frac{b}{S_{b}}\frac{e^{*}}{S_{c}}}}\\ & & +\underset{\text{Prolif.}\;\text{of}\;\text{a}\;\text{mut.}\;S_{b}\;\text{into}\;\text{two}\;\text{mut.}}{\underset{︸}{\frac{r(b^{*}-1)}{r(b^{*}-1)+b}\left(1-u_{2}\right)}}\overset{\text{Migrations:}\;\text{a}\;\text{w.t.}\;S_{b}\;\text{to}\;S_{c}\;\text{and}\;\text{a}\;\text{mut.}\;S_{c}\;\text{to}\;S_{b}}{\overset{︷}{\alpha \frac{b}{S_{b}}\frac{e^{*}}{S_{c}}}})\\ & & +\underset{\text{Diff.}\;\text{of}\;\text{a}\;\text{w.t.}\;S_{b}}{\underset{︸}{\sigma \frac{b}{rb^{*}+b}}}[\left(1-\gamma \right)\overset{\text{Prolif.}\;\text{of}\;\text{a}\;\text{w.t.}\;S_{b}\;\text{into}\;\text{one}\;\text{w.t.}\;\text{and}\;\text{one}\;\text{mut.}}{\overset{︷}{\frac{b-1}{rb^{*}+\left(b-1\right)}\left(1-u_{1}\right)}}\underset{\begin{array}{c} \text{Migrations:}\;\text{a}\;\text{w.t.}\;S_{b}\;\text{to}\;S_{c}\\ \;\text{and}\;\text{a}\;\text{mut.}\;S_{c}\;\text{to}\;S_{b} \end{array}}{\underset{︸}{\alpha \frac{b}{S_{b}}\frac{e^{*}}{S_{c}}}}\\ & & +\gamma \overset{\begin{array}{c} \text{Prolif.}\;\text{of}\;\text{a}\;\text{w.t.}\;S_{c}\;\text{into}\;\text{two}\;\text{w.t.}\\ \text{and}\;\text{a}\;\text{mig.}\;\text{of}\;\text{a}\;\text{mut.}\;S_{c}\;\text{to}\;S_{b} \end{array}}{\overset{︷}{\frac{e}{re^{*}+e}\left(1-u_{1}\right)\frac{e^{*}}{S_{c}}}}]\\ & & \approx \sigma \frac{e^{*}}{S_{c}}\left(\left(1-\gamma \right)\alpha +\gamma \right) \end{array}$$ (8)

The probability that the number of mutant *S*_*b*_ cells stays the same while the number of mutant *S*_*c*_ cells increases by one:

$$\begin{array}{ccc} p_{\left(e^{*},b^{*}\right)\to \left(e^{*}+1,b^{*}\right)} & = & \underset{\text{Diff.}\;\text{of}\;\text{a}\;\text{w.t.}\;S_{b}}{\underset{︸}{\sigma \frac{b}{rb^{*}+b}}}[\left(1-\gamma \right)(\underset{\begin{array}{c} \text{Prolif.}\;\text{of}\;\text{a}\;\text{w.t.}\;S_{b}\;\text{into}\\ \;\text{one}\;\text{w.t.}\;\text{and}\;\text{one}\;\text{mut.} \end{array}}{\underset{︸}{\frac{b-1}{rb^{*}+\left(b-1\right)}u_{1}}}\overset{\begin{array}{c} \text{Migrations:}\;\text{a}\;\text{mut.}\;S_{b}\;\text{to}\;S_{c}\\ \;\text{and}\;\text{a}\;\text{w.t.}\;S_{c}\;\text{to}\;S_{b} \end{array}}{\overset{︷}{\alpha \frac{b^{*}}{S_{b}}\frac{e}{S_{c}}}}\\ & & +\underset{\text{Prolif.}\;\text{of}\;\text{a}\;\text{mut.}\;S_{b}\;\text{into}\;\text{two}\;\text{mut.}}{\underset{︸}{\frac{rb^{*}}{rb^{*}+\left(b-1\right)}\left(1-u_{2}\right)}}\overset{\text{Migrations:}\;\text{a}\;\text{mut.}\;S_{b}\;\text{to}\;S_{c}\;\text{and}\;\text{a}\;\text{w.t.}\;S_{c}\;\text{to}\;S_{b}}{\overset{︷}{\alpha \frac{b^{*}+1}{S_{b}}\frac{e}{S_{c}}}})\\ & & +\gamma (\underset{\text{Prolif.}\;\text{of}\;\text{a}\;\text{w.t.}\;S_{c}\;\text{into}\;\text{one}\;\text{w.t.}\;\text{and}\;\text{one}\;\text{mut.}\;\text{and}\;\text{mig.}\;\text{of}\;\text{a}\;\text{w.t.}\;S_{c}\;\text{to}\;S_{b}}{\underset{︸}{\frac{e}{re^{*}+e}u_{1}\frac{e}{S_{c}}}}+\overset{\text{Prolif.}\;\text{of}\;\text{a}\;\text{mut.}\;S_{c}\;\text{into}\;\text{two}\;\text{mut.}\;\text{and}\;\text{mig.}\;\text{of}\;\text{a}\;\text{w.t.}\;S_{c}\;\text{to}\;S_{b}}{\overset{︷}{\frac{re^{*}}{re^{*}+e}\left(1-u_{2}\right)\frac{e}{S_{c}}}})]\\ & & \approx \sigma \left(1-\gamma \right)u_{1}\alpha \frac{b^{*}}{S_{b}}+\sigma \gamma \left(\frac{re^{*}}{S_{c}}\left(1-u_{2}\right)+u_{1}\right) \end{array}$$ (9)

The probability that the number of mutant *S*_*b*_ cells increases by two while the number of mutant *S*_*c*_ cells decreases by one:

$$\begin{array}{ccc} p_{\left(e^{*},b^{*}\right)\to \left(e^{*}-1,b^{*}+2\right)} & = & \underset{\text{Diff.}\;\text{of}\;\text{a}\;\text{w.t.}\;S_{b}}{\underset{︸}{\sigma \frac{b}{rb^{*}+b}}}\left(1-\gamma \right)(\underset{\begin{array}{c} \text{Prolif.}\;\text{of}\;\text{a}\;\text{w.t.}\;S_{b}\;\text{into}\\ \;\text{one}\;\text{w.t.}\;\text{and}\;\text{one}\;\text{mut.} \end{array}}{\underset{︸}{\frac{b-1}{rb^{*}+\left(b-1\right)}u_{1}}}\overset{\begin{array}{c} \text{Migrations:}\;\text{a}\;\text{w.t.}\;S_{b}\;\text{to}\;S_{c}\\ \;\text{and}\;\text{a}\;\text{mut.}\;S_{c}\;\text{to}\;S_{b} \end{array}}{\overset{︷}{\alpha \frac{b-1}{S_{b}}\frac{e^{*}}{S_{c}}}}\\ & & +\underset{\text{Prolif.}\;\text{of}\;\text{a}\;\text{mut.}\;S_{b}\;\text{into}\;\text{two}\;\text{mut.}}{\underset{︸}{\frac{rb^{*}}{rb^{*}+\left(b-1\right)}\left(1-u_{2}\right)}}\overset{\text{Migrations:}\;\text{a}\;\text{w.t.}\;S_{b}\;\text{to}\;S_{c}\;\text{and}\;\text{a}\;\text{mut.}\;S_{c}\;\text{to}\;S_{b}}{\overset{︷}{\alpha \frac{b-1}{S_{b}}\frac{e^{*}}{S_{c}}}})\\ & & \approx \sigma \left(1-\gamma \right)u_{1}\alpha \frac{e^{*}}{S_{c}} \end{array}$$ (10)

The probability that the number of mutant *S*_*b*_ cells decreases by two while the number of mutant *S*_*c*_ cells increases by one:

$$\begin{array}{ccc} p_{\left(e^{*},b^{*}\right)\to \left(e^{*}+1,b^{*}-2\right)} & = & \underset{\text{Diff.}\;\text{of}\;\text{a}\;\text{mut.}\;S_{b}}{\underset{︸}{\sigma \frac{rb^{*}}{rb^{*}+b}}}\left(1-\gamma \right)\underset{\text{Prolif.}\;\text{of}\;\text{a}\;\text{w.t.}\;S_{b}\;\text{into}\;\text{two}\;\text{w.t.}}{\underset{︸}{\frac{b}{r(b^{*}-1)+b}\left(1-u_{1}\right)}}\\ & & \times \overset{\text{Migrations:}\;\text{a}\;\text{mut.}\;S_{b}\;\text{to}\;S_{c}\;\text{and}\;\text{a}\;\text{w.t.}\;S_{c}\;\text{to}\;S_{b}}{\overset{︷}{\alpha \frac{b^{*}-1}{S_{b}}\frac{e}{S_{c}}}}\approx 0 \end{array}$$ (11)

The probability to create a double-hit mutant in the *S*_*b*_ compartment:

$$\begin{array}{ccc} p_{\left(e^{*},b^{*}\right)\to \left(b^{**}\right)} & = & \underset{\text{Diff.}\;\text{of}\;\text{a}\;\text{w.t.}\;S_{b}}{\underset{︸}{\sigma \frac{b}{rb^{*}+b}}}[\left(1-\gamma \right)\underset{\begin{array}{c} \text{Prolif.}\;\text{of}\;\text{a}\;\text{mut.}\;S_{b}\;\text{into}\\ \;\text{one}\;\text{mut.}\;\text{and}\;\text{one}\;\text{2-hit}\;\text{mut.} \end{array}}{\underset{︸}{\frac{rb^{*}}{rb^{*}+\left(b-1\right)}u_{2}}}\left(\overset{\text{No}\;\text{migration}}{\overset{︷}{\left(1-\alpha \right)}}+\overset{\begin{array}{c} \text{All}\;\text{migrations}\;\text{less}\;\text{mig.}\;\text{of}\\ \;\text{2-hit}\;\text{mut.}\;S_{b}\;\text{to}\;S_{c} \end{array}}{\overset{︷}{\alpha (1-\frac{1}{S_{b}})}}\right)\\ & & +\gamma \underset{\begin{array}{c} \text{Prolif.}\;\text{of}\;\text{mut.}\;S_{c}\;\text{into}\;\text{one}\;\text{mut.}\;\text{and}\;\text{one}\;\text{2-hit}\;\text{mut.}\\ \text{and}\;\text{mig.}\;\text{of}\;\text{2-hit}\;\text{mut.}\;\text{to}\;S_{b} \end{array}}{\underset{︸}{\frac{re^{*}}{re^{*}+e}u_{2}\frac{1}{S_{c}}}}]\\ & & +\underset{\text{Diff.}\;\text{of}\;\text{a}\;\text{mut.}\;S_{b}}{\underset{︸}{\sigma \frac{rb^{*}}{rb^{*}+b}}}[\left(1-\gamma \right)\underset{\begin{array}{c} \text{Prolif.}\;\text{of}\;\text{a}\;\text{mut.}\;S_{b}\;\text{into}\;\text{one}\;\text{mut.}\\ \;\text{and}\;\text{one}\;\text{2-hit}\;\text{mut.} \end{array}}{\underset{︸}{\frac{r(b^{*}-1)}{r(b^{*}-1)+b}u_{2}}}\left(\overset{\text{No}\;\text{migration}}{\overset{︷}{\left(1-\alpha \right)}}+\overset{\begin{array}{c} \text{All}\;\text{migrations}\;\text{less}\;\text{mig.}\;\text{of}\\ \;\text{2-hit}\;\text{mut.}\;S_{b}\;\text{to}\;S_{c} \end{array}}{\overset{︷}{\alpha (1-\frac{1}{S_{b}})}}\right)\\ & & +\gamma \overset{\begin{array}{c} \text{Prolif.}\;\text{of}\;\text{mut.}\;S_{c}\;\text{into}\;\text{one}\;\text{mut.}\;\text{and}\;\text{one}\;\text{2-hit}\;\text{mut.}\\ \text{and}\;\text{mig.}\;\text{of}\;\text{2-hit}\;\text{mut.}\;\text{to}\;S_{b} \end{array}}{\overset{︷}{\frac{re^{*}}{re^{*}+e}u_{2}\frac{1}{S_{c}}}}]+\underset{\begin{array}{c} \text{two}\;\text{asym.}\;\text{mut.}\;S_{b}\;\text{divisions}\\ \;\text{with}\;\text{2-hit}\;\text{mut.}\;\text{in}\;S_{b} \end{array}}{\underset{︸}{2\left(1-\sigma \right)\frac{rb^{*}}{rb^{*}+b}u_{2}\nu }}\\ & & \approx \sigma \frac{rb^{*}}{S_{b}}\left(1-\gamma \right)u_{2}+2\left(1-\sigma \right)\frac{rb^{*}}{S_{b}}u_{2}\nu \end{array}$$ (12)

The probability to create a double-hit mutant in the *S*_*c*_ compartment:

$$\begin{array}{ccc} p_{\left(e^{*},b^{*}\right)\to \left(e^{**}\right)} & = & \underset{\text{Diff.}\;\text{of}\;\text{a}\;\text{w.t.}\;S_{b}}{\underset{︸}{\sigma \frac{b}{rb^{*}+b}}}\left(1-\gamma \right)\underset{\begin{array}{c} \text{Prolif.}\;\text{of}\;\text{a}\;\text{mut.}\;S_{b}\;\text{into}\\ \text{one}\;\text{mut.}\;\text{and}\;\text{one}\;\text{2-hit}\;\text{mut.} \end{array}}{\underset{︸}{\frac{rb^{*}}{rb^{*}+\left(b-1\right)}u_{2}}}\overset{\text{Migration}\;\text{of}\;\text{2-hit}\;\text{mut.}\;\text{to}\;S_{c}}{\overset{︷}{\alpha \frac{1}{S_{b}}}}\\ & & +\underset{\text{Diff.}\;\text{of}\;\text{a}\;\text{w.t.}\;\text{or}\;\text{mut.}\;S_{b}}{\underset{︸}{\sigma \frac{rb^{*}+b}{rb^{*}+b}}}\gamma \overset{\begin{array}{c} \text{Prolif.}\;\text{of}\;\text{a}\;\text{mut.}\;S_{c}\;\text{into}\;\text{one}\;\text{mut.}\;\text{and}\;\text{one}\;\text{2-hit}\;\text{mut.}\\ \;\text{and}\;\text{all}\;\text{mig.}\;\text{less}\;\text{mig.}\;\text{of}\;\text{2-hit}\;\text{mut.}\;\text{to}\;S_{b} \end{array}}{\overset{︷}{\frac{re^{*}}{re^{*}+e}u_{2}\left(1-\frac{1}{S_{c}}\right)}}\\ & & +\underset{\text{Diff.}\;\text{of}\;\text{a}\;\text{mut.}\;S_{b}}{\underset{︸}{\sigma \frac{rb^{*}}{rb^{*}+b}}}\left(1-\gamma \right)\underset{\begin{array}{c} \text{Prolif.}\;\text{of}\;\text{mut.}\;S_{b}\;\text{into}\;\text{one}\;\text{mut.}\\ \;\text{and}\;\text{one}\;\text{2-hit}\;\text{mut.} \end{array}}{\underset{︸}{\frac{r(b^{*}-1)}{r(b^{*}-1)+b}u_{2}}}\overset{\text{Migration}\;\text{of}\;\text{2-hit}\;\text{mut.}\;\text{to}\;S_{c}}{\overset{︷}{\alpha \frac{1}{S_{b}}}}\\ & & \approx \sigma \gamma \frac{re^{*}}{S_{c}}u_{2} \end{array}$$ (13)

The probability to create a double-hit mutant:

$$\begin{array}{c} p_{(e^{*},b^{*})\to E}\approx \sigma ru_{2}\left(\frac{b^{*}}{S_{b}}\left(1-\gamma \right)+\frac{e^{*}}{S_{c}}\gamma \right)+2\left(1-\sigma \right)\frac{rb^{*}}{S_{b}}u_{2}\nu \end{array}$$ (14)

We can define *φ*_e*, b*_(*t*) as the probability to have e* mutants in the *S*_*c*_ compartment and b* mutants in the *S*_*b*_ compartment at time *t*. Using the transition probabilities at time *t* − 1, the Kolmogorov forward equation for the function is given by:

$$\begin{array}{ccc} \dot{\varphi } & = & [\sigma \frac{1-\gamma }{S_{b}}\left(r\left(b^{*}-1\right)\left(1-u_{2}\right)+S_{b}u_{1}\right)+\sigma \gamma u_{1}\frac{e^{*}}{S_{c}}+2\left(1-\sigma \right)u_{1}\nu ]\varphi \left(e^{*},b^{*}-1\right)\\ & & +\sigma \frac{r(b^{*}+1)}{S_{b}}\left(1-u_{1}\right)\varphi \left(e^{*},b^{*}+1\right)\\ & & +\sigma \frac{b^{*}+1}{S_{b}}\left(1-\gamma \right)\alpha \left(1-u_{1}\right)\varphi \left(e^{*}-1,b^{*}+1\right)\\ & & +\sigma \frac{e^{*}+1}{S_{c}}\left(\left(1-\gamma \right)\alpha +\gamma \right)\varphi \left(e^{*}+1,b^{*}-1\right)\\ & & +\sigma [\left(1-\gamma \right)u_{1}\alpha \frac{b^{*}}{S_{b}}+\gamma \left(\frac{r(e^{*}-1)}{S_{c}}\left(1-u_{2}\right)+u_{1}\right)]\varphi \left(e^{*}-1,b^{*}\right)\\ & & +\sigma \frac{e^{*}+1}{S_{c}}\left(1-\gamma \right)\alpha u_{1}\varphi \left(e^{*}+1,b^{*}-2\right)\\ & & -[\sigma (u_{1}+\frac{rb^{*}}{s_{b}}\left(2-\gamma -u_{1}\right)+\frac{b^{*}}{S_{b}}\left(1-\gamma \right)\alpha +\frac{\gamma e^{*}}{S_{c}}\left(1+r+\frac{1-\gamma }{\gamma }\alpha \left(1-u_{1}\right)+u_{1}\right))\\ & & +2\left(1-\sigma \right)\nu \left(u_{1}+\frac{rb^{*}}{S_{b}}u_{2}\right)]\varphi \left(e^{*},b^{*}\right) \end{array}$$ (15)

We define the probability generating function by:

$$\begin{array}{ccc} \psi (x,y,;t) & = & \sum_{e^{*},b^{*}}\varphi_{e^{*},b^{*}}\left(t\right)x^{b^{*}}y^{e^{*}} \end{array}$$ (16)

The probability to be in one of the states (e*, b*) is given by *ψ*(1, 1; *t*). Therefore, the probability to transition to state E is *P*_2_(*t*) = 1 − *ψ*(1, 1; *t*). The probability generating function gives the following equations:

$$\begin{array}{ccc} \frac{\delta \psi }{\delta t} & = & \frac{\sigma \gamma }{s_{c}}\frac{\delta \psi }{\delta y}(\frac{1-\gamma }{\gamma }\alpha u_{1}x^{2}+\left(1+\frac{1-\gamma }{\gamma }\alpha \right)x+r\left(1-u_{2}\right)y^{2}+u_{1}xy\\ & & -(r+1+u_{1}+\frac{1-\gamma }{\gamma }\alpha \left(1+u_{1}\right))y)\\ & & +\frac{r}{S_{b}}\frac{\delta \psi }{\delta x}(\sigma [\left(1-\gamma \right)\left(1-u_{2}\right)x^{2}-\left(2-\gamma -u_{1}+\frac{(1-\gamma )\alpha }{r}\right)x+\frac{(1-\gamma )\alpha }{r}u_{1}xy\\ & & +\frac{(1-\gamma )\alpha }{r}\left(1-u_{1}\right)y+1-u_{1}]-2\left(1-\sigma \right)u_{2}\nu x)\\ & & -u_{1}\psi (\sigma \gamma (1-y)+(\sigma (1-\gamma )+2(1-\sigma )\nu )(1-x)) \end{array}$$ (17)

We have,

$$\begin{array}{ccc} P_{2}\left(t\right) & = & 1-exp(-u_{1}\int_{0}^{t}[\sigma \gamma \left(1-y\left(t^{\prime }\right)\right)+\left(\sigma \left(1-\gamma \right)+2\left(1-\sigma \right)\nu \right)\\ & & \times \left(1-x\left(t^{\prime }\right)\right)]dt^{\prime }) \end{array}$$ (18)

where

$$\begin{array}{ccc} \dot{y} & = & \frac{\sigma \gamma }{S_{c}}(x+r\left(1-u_{2}\right)y^{2}-\left(r+1\right)y+\frac{1-\gamma }{\gamma }\alpha \left(x-y\right)), \end{array}$$ (19)

$$\begin{array}{ccc} \dot{x} & = & \frac{r}{S_{b}}(\sigma [\left(1-\gamma \right)\left(1-u_{2}\right)x^{2}+\left(1-\gamma \right)\frac{\alpha }{r}\left(\left(1-u_{1}\right)y+u_{1}xy-x\right)\\ & & -x\left(2-\gamma -u_{1}\right)+1-u_{1}]-2\left(1-\sigma \right)u_{2}\nu x)\\ & & \approx \frac{r}{S_{1}}(\sigma \left[\left(1-\gamma \right)\left(1-u_{2}\right)x^{2}+\left(1-\gamma \right)\frac{\alpha }{r}\left(y-x\right)-\left(2-\gamma \right)x+1\right]\\ & & -2\left(1-\sigma \right)u_{2}\nu x) \end{array}$$ (20)

## Tunneling conditions

Here, we investigate the applicability of the tunneling rate, for more information please see [41]. The tunneling rate is practical when one-hit mutants exist at low numbers, and the creation of a two-hit mutant occurs without prior fixation of 1-hit mutants. A condition for such a scenario is that the fixation rate for one-hit mutants is smaller than the tunneling rate.

Let us denote the probability of fixation of 1-hit mutants in the *S*_*b*_ compartment (or in the *S*_*c*_ group), starting from the state with b* number of BSC mutants (or e_*_ number of CeSC mutants) by $\pi_{b^{*}}^{S_{b}}$ (or $\pi_{e^{*}}^{S_{c}}$). Then tunneling occurs if the probability of absorbing in the state of all one-hit mutants (when there is no chance of new mutations i.e *u*_1_ = *u*_2_ = 0) is less than the probability of creation of new mutation, i.e. $\pi_{b^{*}}^{S_{b}}\;\text{and}\;\pi_{e^{*}}^{S_{c}}⪡u_{2}$.

The quantities $\pi_{b^{*}}^{S_{b}}$ and $\pi_{e^{*}}^{S_{c}}$ satisfy the following systems:

$$\begin{array}{ccc} \pi_{b^{*}}^{S_{b}} & = & P_{b^{*}\to S_{b}}+\sum_{m=1}^{S_{b}-1}P_{b^{*}\to m}\pi_{m}^{S_{b}} \end{array}$$ (21)

$$\begin{array}{ccc} \pi_{e^{*}}^{S_{c}} & = & P_{e^{*}\to S_{c}}+\sum_{m=1}^{S_{c}-1}P_{e^{*}\to m}\pi_{m}^{S_{c}} \end{array}$$ (22)

For more details about obtaining fixation probability please see [42]. We first calculate the probability of transforming from a one-hit mutant to the all 1-hit mutant state by assuming no new mutation will be created, i.e. *u*_1_ = *u*_2_ = 0. Therefore, the non-zero transition probabilities are

$$\begin{array}{ccc} & & P_{\left(e^{*},b^{*}\right)\to \left(e^{*},b^{*}+1\right)}\approx \sigma \left(1-\gamma \right)\frac{rb^{*}}{S_{b}}\\ & & P_{\left(e^{*},b^{*}\right)\to \left(e^{*},b^{*}-1\right)}\approx \frac{\sigma rb^{*}}{S_{b}}\\ & & P_{\left(e^{*},b^{*}\right)\to \left(e^{*}+1,b^{*}-1\right)}\approx \sigma \left(1-\gamma \right)\alpha \frac{b^{*}}{S_{b}}\\ & & P_{\left(e^{*},b^{*}\right)\to \left(e^{*}-1,b^{*}+1\right)}\approx \sigma \frac{e^{*}}{S_{c}}\left(\left(1-\gamma \right)\alpha +\gamma \right)\\ & & P_{\left(e^{*},b^{*}\right)\to \left(e^{*}+1,b^{*}\right)}\approx \sigma \gamma \frac{re^{*}}{S_{c}} \end{array}$$ (23)

We calculate the probability of transformation from a single one-hit mutant in the *S*_*b*_ compartment (i.e. (e*, b*) = (0, 1)) to all one-hit mutants in the *S*_*b*_ group (b* = *S*_*b*_). By substituting the transition probabilities Eq (23) in Eq (21), we get

$$\begin{array}{ccc} & & r\left(1-\gamma \right)\pi_{b^{*}+1}^{S_{b}}+\left(r+\left(1-\gamma \right)\alpha \right)\pi_{b^{*}-1}^{S_{b}}-\left(r+\left(1-\gamma \right)\left(\alpha +r\right)\right)\pi_{b^{*}}^{S_{b}}=0\;\;\;1<b^{*}<S_{b}\\ & & r\left(1-\gamma \right)\pi_{2}^{S_{b}}-\left(r+\left(1-\gamma \right)\left(\alpha +r\right)\right)\pi_{1}^{S_{b}}=0\\ & & r\left(1-\gamma \right)+\left(r+\left(1-\gamma \right)\alpha \right)\pi_{S_{b}-2}^{S_{b}}-\left(r+\left(1-\gamma \right)\left(\alpha +r\right)\right)\pi_{S_{b}-1}^{S_{b}}=0 \end{array}$$ (24)

This system implies

$$\begin{array}{c} \pi_{b^{*}}^{S_{b}}=\frac{1-{\left(\frac{r+(1-\gamma )\alpha }{r(1-\gamma )}\right)}^{b^{*}}}{1-{\left(\frac{r+(1-\gamma )}{r(1-\gamma )}\right)}^{S_{b}}} \end{array}$$ (25)

Thus, the fixation probability of moving from the state that has only a single BSC mutant to the state that all BSCs are mutants is given by

$$\begin{array}{c} \pi_{1}^{S_{b}}=\frac{1-(\frac{r+(1-\gamma )\alpha }{r(1-\gamma )})}{1-{\left(\frac{r+(1-\gamma )}{r(1-\gamma )}\right)}^{S_{b}}} \end{array}$$ (26)

Additionally, we obtain the probability of transformation from single one-hit mutant in the *S*_*c*_ compartment (i.e. (e*, b*) = (1, 0)) to the state that all CeSCs are one-hit mutants (e* = *S*_*c*_). From Eqs (22) and (23) we have

$$\begin{array}{c} r\gamma \pi_{e^{*}+1}^{S_{c}}+\left(\gamma +\left(1-\gamma \right)\alpha \right)\pi_{e^{*}-1}^{S_{c}}-\left(\gamma \left(r+1\right)+\left(1-\gamma \right)\alpha \right)\pi_{e^{*}}^{S_{c}}=0\;\;\;1<e^{*}<S_{c} \end{array}$$ (27)

$$\begin{array}{c} r\gamma \pi_{2}^{S_{c}}-\left(\gamma \left(r+1\right)+\left(1-\gamma \right)\alpha \right)\pi_{1}^{S_{c}}=0 \end{array}$$ (28)

$$\begin{array}{c} r\gamma +\left(\gamma +\left(1-\gamma \right)\alpha \right)\pi_{S_{c}-2}^{S_{c}}-\left(\gamma \left(r+1\right)+\left(1-\gamma \right)\alpha \right)\pi_{S_{c}-1}^{S_{c}}=0 \end{array}$$ (29)

This implies

$$\begin{array}{ccc} \pi_{e^{*}}^{S_{c}} & = & \frac{1-{\left(\frac{\gamma +(1-\gamma )\alpha }{r\gamma }\right)}^{e^{*}}}{1-{\left(\frac{\gamma +(1-\gamma )\alpha }{r\gamma }\right)}^{S_{c}}} \end{array}$$ (30)

$$\begin{array}{ccc} \pi_{1}^{S_{c}} & = & \frac{1-(\frac{\gamma +(1-\gamma )\alpha }{r\gamma })}{1-{\left(\frac{\gamma +(1-\gamma )\alpha }{r\gamma }\right)}^{S_{c}}} \end{array}$$ (31)

Thus in the second model we have tunneling if

$$\begin{array}{c} \max\left(\frac{1-(\frac{\gamma +(1-\gamma )\alpha }{r\gamma })}{1-{\left(\frac{\gamma +(1-\gamma )\alpha }{r\gamma }\right)}^{S_{c}}},\frac{1-(\frac{r+(1-\gamma )\alpha }{r(1-\gamma )})}{1-{\left(\frac{r+(1-\gamma )}{r(1-\gamma )}\right)}^{S_{b}}}\right)<u_{2} \end{array}$$ (32)
